# Development of dialogue system architecture toward co-creating social intelligence when talking with a partner robot

**DOI:** 10.3389/frobt.2022.933001

**Published:** 2022-10-17

**Authors:** Ayaka Fujii, Kristiina Jokinen, Kei Okada, Masayuki Inaba

**Affiliations:** ^1^ AI Research Center, National Institute of Advanced Industrial Science and Technology, Tokyo, Japan; ^2^ Graduate School of Information Science and Technology, The University of Tokyo, Tokyo, Japan

**Keywords:** human–robot interaction, conversational agent, co-creating, system integration, social robot

## Abstract

Social robots have grown increasingly integrated into our daily lives in recent years. Robots can be good social agents who engage with people, such as assistants and counselors, and good partners and companions with whom people can form good relationships. Furthermore, unlike devices such as smart speakers or virtual agents on a screen, robots have physicality, which allows them to observe the actual environment using sensors and respond behaviorally with full-body motions. In order to engage people in dialogue and create good relationships with robots as close partners, real-time interaction is important. In this article, we present a dialogue system platform developed with the aim of providing robots with social skills. We also built a system architecture for the robot to respond with speech and gestures within the dialogue system platform, which attempts to enable natural engagement with the robot and takes advantage of its physicality. In addition, we think the process called “co-creation” is important to build a good human–robot interaction system. Engineers must bridge the gap between users and robots in order for them to interact more effectively and naturally, not only by building systems unilaterally but also from a range of views based on the opinions of real users. We reported two experiments using the developed dialogue interaction system with a robot. One is an experiment with elderly people as the initial phase in this co-creation process. The second experiment was conducted with a wide range of ages, from children to adults. Through these experiments, we can obtain a lot of useful insights for improving the system. We think that repeating this co-creation process is a useful approach toward our goal that humans and robots can communicate in a natural way as good partners such as family and friends.

## 1 Introduction

In recent years, robots have become more and more closely related to our daily lives. While production sites such as factories have traditionally been the main places where robots have been active, recently they have been working in social roles. Robots are not only useful machines but also social agents that interact with humans, such as assistants who respond to human requests and give advice (Kanda et al., 2010) and partners with whom we can feel a psychological connection (Samani et al., 2010). In addition, robots have physicality; they can observe the real world using various sensors and respond behaviorally using full-body gestures, which is different from smart speakers or virtual agents on a screen.

In order for robots to become better social beings, they must not only move according to programs predetermined by humans but also change their behavior in real time through interaction with humans (Jokinen and Wilcock, 2021). Interacting with robots can be done in a variety of ways. For example, tablets and keyboards are useful input interfaces. People can also interact with robots by using gestures and eye-tracking (Berg et al., 2019). However, among many interaction methods, voice interaction, which is the most common way for humans to communicate with each other, is considered to be essential for robots to naturally integrate into human society (Jokinen, 2009). We can communicate by voice even when both hands are occupied or when we are out of sight of the robot.

We think three main technologies are important in order to have conversational interaction with a robot: speech recognition technology that converts speech data into text data, natural language processing technology that understands what the text data is intended to say, and dialogue management technology that determines the appropriate speech content and gestures that the robot should make in response to the intentions. With the development of artificial intelligence research in recent years, there has been remarkable progress in each of these three technologies, but there is almost no open-sourced system that integrates these technologies in a way that is easy for robots to use. In this article, we propose a dialogue system platform that aims to serve as a basis for the robot to acquire social skills. In the dialogue system platform, we have also developed a system architecture that enables the robot to respond not only by speech but also by gestures, taking advantage of the physicality of the robot.

In addition, in order for humans and robots to better interact, engineers must bridge the gap that exists between robots and humans, not only by developing systems unilaterally but also from a variety of perspectives based on the opinions of actual users. We think the process called “co-creation” is important. Co-creation is a method that considers users as partners in creating new systems and values, and extracts and solves issues through discussions with the users. We also discuss this co-creation process and describe some experiments on robot interaction in this article.

This article is organized as follows. Section 2 shows the related technologies and we explain the system architecture of the dialogue system platform in Section 3. Section 4 describes and discusses two small experiments using the developed dialogue system, and argues the method of co-creating. Finally, Section 5 concludes and discusses future work. The main contribution of this article is building the total dialogue system architecture from speech recognition to robot’s response expression by speaking and gesturing in the open-source format and conducting experiments with diverse age people in the mealtime situation which is a very common activity but the new research field in human–robot interaction.

## 2 Related works

In this section, examples of social robot applications and the basic technologies for dialogue interaction with robots will be described.

### 2.1 Social robot application

Recently, robots play an active role in various fields of society. In Japan, Pepper (SoftBank Robotics) can sometimes be seen in stores giving information and working as a receptionist. Robots also serve as waiters in restaurants (Cheong et al., 2016), hotel guides (Pinillos et al., 2016), and airport guides (Triebel et al., 2016). Furthermore, robot usage in general households has been remarkable. Not only robot vacuum cleaners such as Roomba (iRobot), but also robots that act as daily assistants such as Sota (Vstone) and Romi (mixi), and pet-like robots that cannot speak such as AIBO (SONY) and LOVOT (GROOVE X) are gradually becoming popular in homes. In addition, at the research level, robots are developed to perform diverse roles in society and the home, such as mental coaching (Jeong et al., 2020), driving a car (Paolillo et al., 2017), cooking (Liu et al., 2022), and folding clothes (Yaqiang et al., 2018). However, though there is some research on robots that assist in eating (Naotunna et al., 2015), there is little research on robots as communication partners during meals. We think that mealtime is an important time at which most people spend several hours each day, and robots have great potential to be good companions to eat with. Therefore, in this article, we conducted experiments on interaction with a robot in the setting of a meal scene.

We think that robots will become more common in society in the future. In Japan, some families treat robots as members of the family, for example, by holding funerals for AIBO (Knox and Watanabe, 2018), and not a few people consider robots as partners who live together. In order for robots to fit better into our society, they need to not only have useful functions but also interact and coexist better with humans. From this point of view, it is necessary to develop the robot while taking into account the opinions of users and considering how the robot should be together. Therefore, in this article, we introduce the co-creation method and conducted the experiments as the first step of the co-creation process.

### 2.2 Dialogue interaction

As mentioned in the introduction, we think three main technologies are important in order to have conversational interaction with a robot: speech recognition, natural language processing, and dialogue management. In this subsection, we will provide a brief summary of the latest technologies in each.

Speech recognition systems, such as Julius (Lee and Kawahara, 2009) and Kaldi (Povey et al., 2011), that combine multiple models for computing probabilities have been developed. In these systems, features are first extracted from the speech waveform and converted into phonemes using acoustic models such as a Hidden Markov Model (HMM). The phonemes are used to recognize words and sentences through word dictionaries and language models represented by N-grams. Recently, there are much research on speech recognition using end-to-end models which integrate these models using deep learning, such as Connectionist Temporal Classification (CTC) (Graves et al., 2006), Attention Encoder-Decoder (Bahdanau et al., 2016), and Transformer (Dong et al., 2018). Speech recognition systems using end-to-end models, such as ESPnet (Watanabe et al., 2018; Karita et al., 2019; Li et al., 2021), produce better recognition results than conventional methods. In addition, there are many speech to text application programming interfaces (API) such as Google Cloud, Microsoft Azure, and Amazon Transcribe. Though there is a slight time lag since these APIs use cloud services, they can be used to conduct speech recognition easily.

Natural language processing involves the recognition of the meaning of a sentence and the context of the dialogue. The morphological analysis is performed at first generally to separate parts of speech such as nouns, verbs, and particles. They are converted into vectors and put into language processing models such as Transformer (Vaswani et al., 2017) and RNNLM (Mikolov et al., 2010). In recent years, models that have been pre-generated by training on large data sets, such as those represented by BERT (Devlin et al., 2018), are often used. In addition, APIs such as Amazon Comprehend and Natural Language by Google are provided.

Dialogue management can be mainly divided into two functions: task-oriented dialogue agent and chatbot (Jokinen and McTear, 2009; Jurafsky and Martin, 2009). Task-oriented dialogue agents use dialogue to do specific tasks such as making restaurant reservations, looking up train schedules, and communicating with online help systems. Chatbots are typically used for unstructured talks such as chatting. They can also be utilized to construct a task-oriented dialogue system in a natural conversational flow. Commercial chatbot frameworks like Amazon Lex and Google DialogueFlow are available, as well as open-source frameworks such as Rasa (Bocklisch et al., 2017; Kong and Wang, 2021) and OpenDial (Lison and Kennington, 2016). They provide the possibility to build beneficial conversation systems for many people. In the recent research concerning chatbots, the system for interacting based on long-term memory which contains information about the user and internet searches (Komeili et al., 2021; Xu et al., 2021), and the system for identifying the user by face recognition and looking up the saved user profile to adapt to the user (Jokinen and Wilcock, 2021) are developed.

The development in each of these technologies for spoken dialogue interaction is notable. However, there are few systems that integrate these technologies in a format that is easy to use with robots. Some spoken dialogue system platform using Robot Operating System (ROS) was developed. For example, rospeex is a system that performs speech recognition and speech synthesis using the cloud (Sugiura and Zettsu, 2015). PRINTEPS is an integrated robot development platform using knowledge-based inference (Morita et al., 2018). However, they are currently not open source and not everyone can use them. Regarding open-source software, ROS4HRI has been developed for human–robot interaction (Mohamed and Lemaignan, 2021). The system uses multimodal information, combining voice recognition with facial recognition and posture recognition to identify people. As for a dialogue interaction, though the component for speech recognition is included, the system does not include the generation of robot responses, and the users need to implement the natural language processing and dialogue management parts individually. In addition to the current lack of open-source integrated spoken dialogue systems for robots, we think it is useful for robots to respond not only by speech but also by gesturing. Therefore, we developed an open-sourced dialogue system platform for the robots (Fujii and Jokinen, 2022).

## 3 Dialogue system platform

### 3.1 Overview of the system

Our system is built on a ROS platform, integrating ESPnet speech recognizer, Rasa dialogue model, and Nao Robot (SoftBank Robotics). ROS is a widely used platform in robotics research, including human–robot interaction. We chose ESPnet among the many open-source speech recognizers because of its usability in a variety of languages and the balance between the processing time and the accuracy of the results. Rasa, an open-source Conversational AI framework, was used for natural language processing and dialogue management. Gestures are also included in the robot’s response, and we link them to speech content in the dialogue framework.

**FIGURE 1:**
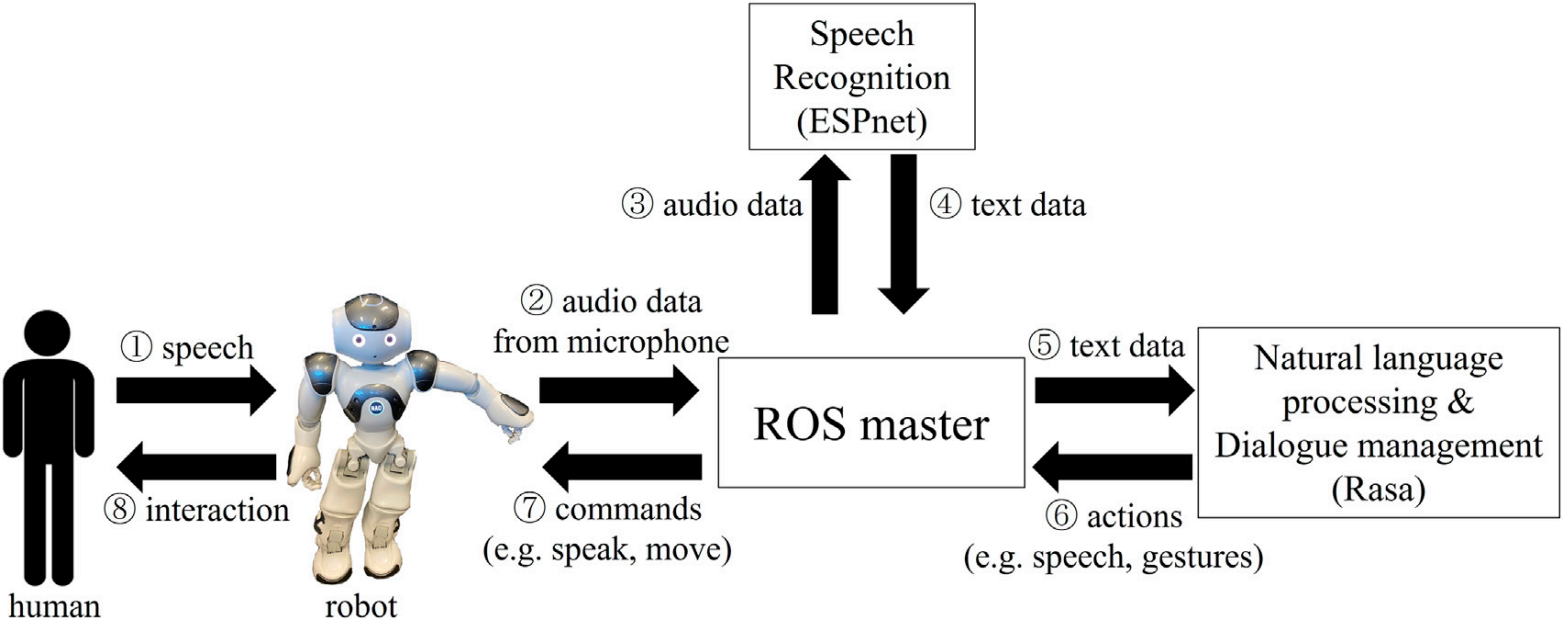
The data flow in the proposed dialogue interaction system that integrates ROS, ESPnet, Rasa, and Nao robots. Modified from Fujii and Jokinen (2022).

The system overview is shown in Figure 1. The sound from the microphone of the robot is sent as rostopic to the laptop running ROS master. The ESPnet speech recognizer converts the audio data to text, which is passed to the Rasa dialogue model. Through Rasa, the user’s intent is classified, and appropriate response actions are chosen based on natural language understanding models and the dialogue policy model. Rasa returns to the next conversational state with speech and gesture actions and the robot moves and speaks in response to the action commands. The code is available on the following website.

https://github.com/aistairc/OpenSource4NaturalHRInteraction.

We use the system in Ubuntu 18.04, ROS melodic, and NAO V6. Each component is described in detail below.

### 3.2 Robot operating system: ROS

ROS is an operating system and tool that is open source for robots. The master system, known as ROS master, and multiple processes, known as nodes, are set up in ROS, and data is communicated between them. There are two methods of communication in ROS: asynchronous communication using topic and synchronous communication using service. We choose topic-based communication because it allows us to send and receive messages continuously in real time. A data-delivery node called the publisher writes a message to a topic, and another node called a subscriber receives the message in topic communication. A message sent by one node can also be received by multiple nodes.

### 3.3 Speech recognizer: ESPnet

ESPnet is an end-to-end speech processing toolkit that is open source (Watanabe et al., 2018; Li et al., 2021). It can perform several language processing tasks, including text-to-speech, speech translation, and machine translation, but in the developed system, we only use the speech recognition function. A script called a recipe, which shows the sequence of data download, preprocessing, feature extraction, training, and model evaluation, ensures reproducibility. In addition, pre-trained models for a variety of languages are provided in the Zenodo community. Though we use a pre-trained model for simplicity (Ando and Fujihara, 2021), fine-tuning can be done based on the application. In the developed system, audio data from the robot’s microphone is recorded in the external laptop for up to 5 seconds before being converted to text data using ESPnet.

### 3.4 Natural language processing and dialogue management: Rasa

Rasa (Bocklisch et al., 2017) is an open-source dialogue framework. It uses cutting-edge machine-learning technology like transformers. The natural language understanding module (NLU Pipeline) and the dialogue management module (Dialogue Policies) are the two main modules that make up this framework. Two shows the system flow of Rasa in our system. It can be optimized for a specific application. The NLU Pipeline can be customized by adding its modules such as tokenizers and entity extractors, or by using external databases. The dialogue management module trains the dialogue model using data called stories and rules, and the model selects the next action based on the user input. A story is a representation of a conversation’s flow, with user inputs represented as intents and system outputs as actions. Rules are used to produce the same output for the same input such as returning greetings when greeted. It is also possible to use *forms* to store data from previous conversations and checkpoints to provide responses based on the current conversational state. Actions can be customized freely and they can also be connected to external databases and services.

**FIGURE 2:**
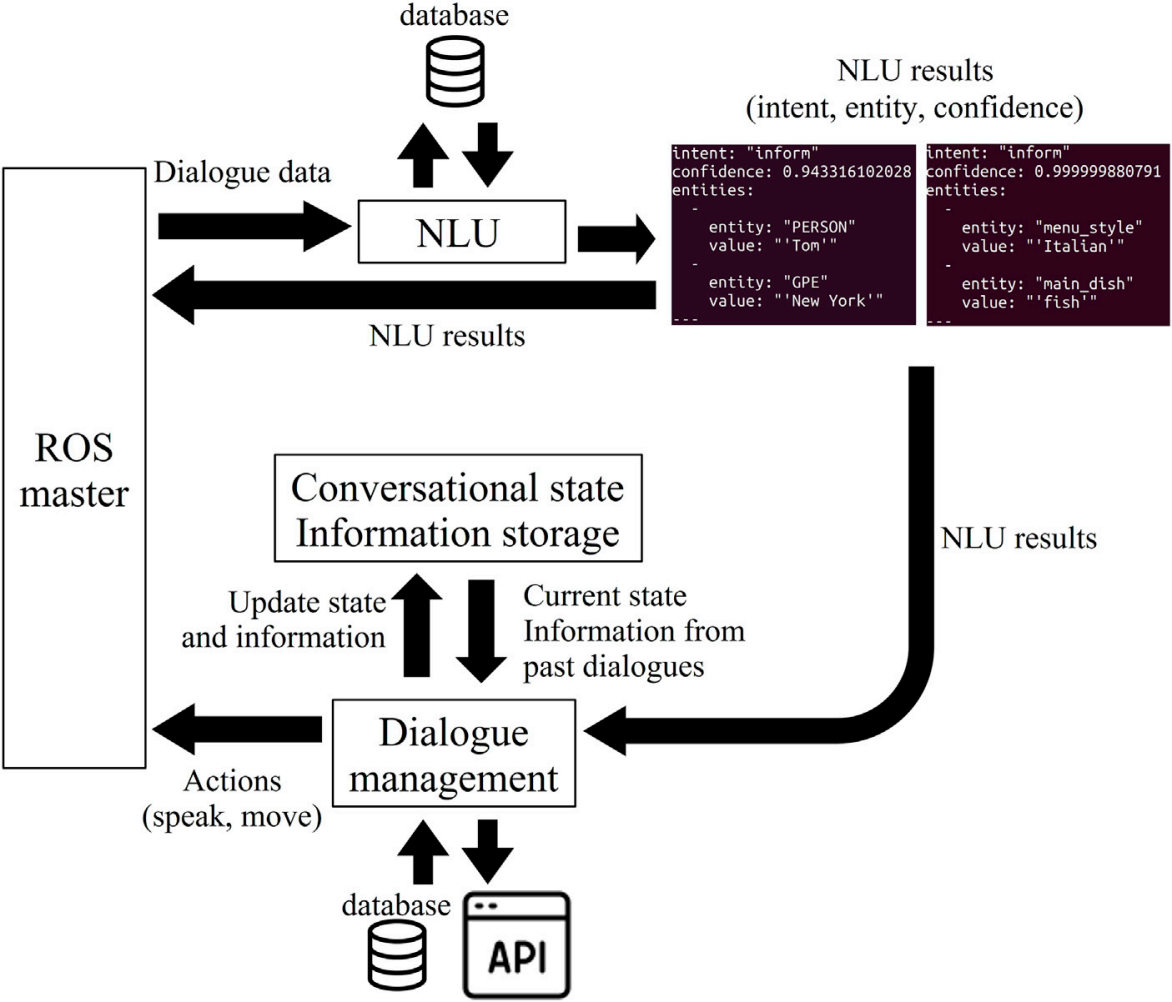
Overview of the natural language processing and dialogue management system using Rasa and ROS. NLU results and the reactions of the robot are returned as rostopics. Modified from Fujii and Jokinen (2022).

In our system, shown in Figure 2, Rasa receives the text data from the speech recognizer as an input message. The intent, confidence, and entities of the input are extracted and published as rostopic to be used in other ROS applications *via* the NLU Pipeline. The dialogue management module also publishes the agent response as rostopic for the speech synthesizer of the robot. The robot’s gesturing is also determined through the dialogue management module and the commands for moving the robot are sent through ROS by using the custom actions.

### 3.5 Robot: NAO

We use the robot NAO (Softbank Robotics Europe). It is a small humanoid robot with 25 degrees of freedom and a height of approximately 58 cm. There are two cameras, two speakers, and four microphones in the head. We chose a microphone with the least noise to use since there are many noise effects from other components in the robot’s head.

The operating system for the NAO robot is NaoqiOS, a Gentoo-based Linux distribution. We use *naoqi_driver* which is an open-sourced software for connecting ROS and NaoqiOS. Though *naoqi_driver* enables ROS to call some of the NAOqi APIs, we created additional rostopic publishers that inherit *NaoqiNode* class from *naoqi_driver*, such as outputting audio data as *AudioData* type topics that are easy to use in ESPnet. In addition, we use the Naoqi APIs’ *ALSoundDetection* function to publish a rostopic that detects human voice and triggers ESPnet to begin speech recognition. We added a function that disables speech recognition when the NAO itself is speaking in order to prevent the misrecognition of the robot’s voice. We use the speech synthesis module that was originally installed in Nao to create the robot’s speech.

## 4 Experiments using the developed dialogue system

In this section, we introduce two human–robot interaction experiments using the developed dialogue system. In these experiments, we could confirm a proof-of-concept of the dialogue system architecture. We also obtain useful opinions and insights towards the future human–robot interaction through communication with participants of the experiments, which we call the “co-creation method.”

### 4.1 Co-creation method

In order for science and technology directly related to our daily lives to be implemented in a way that fits the real world well, it is important not only to improve the technology itself and take approaches to solve the problems of society as a whole but also to understand the demands at the user level. Especially for robots that are not only useful machines but also can have roles as assistants and partners, it is necessary to promote technological development and social implementation from various perspectives, including human science and psychological knowledge. It is also important to build a social foundation to accept new science and technology. In recent years, ethical considerations regarding robots as social beings, such as rights, duties, and responsibilities, have begun to be made (Loh, 2019), but it is also necessary to consider, at the user level, what we would like robots to do and how robots coexist in our society that will come in the future.

We think the “co-creation” method is meant to achieve good social implementation of robots. Co-creation is a method of identifying and resolving issues through discussions with users, viewing them as partners in creating new systems and values. In addition, as users interact with the robots through the co-creation process, they can imagine their future life with robots and think about the role and presence of robots and their relationship with robots. By repeating this process of co-creation over and over again, we believe that robots that can coexist more naturally and better with humans can be realized. This article describes two experiments conducted on people of various ages using the proposed dialogue system as an initial step in the co-creation process, which aims to collect opinions from various people and improve the robot system for social implementation.

### 4.2 Situation setting

We chose mealtime interaction as the situation setting of the experiments. It is thought that the mealtime situation is a good application for voice interaction with the robot, since using input interfaces such as a tablet when both hands tend to be occupied with cutlery and dishes is inconvenient and unclean. Furthermore, due to the social structure changes such as the increase in the number of people living alone in recent years and the spread of infectious diseases, opportunities for sharing mealtime with other people are declining. It is known that eating with others enriches our meal experience and improves life satisfaction and quality of life (Choi et al., 2020; Kim, 2020). We think eating with a robot partner can be a good alternative. However, there is little research on human–robot spoken dialogue interaction in eating situations. Therefore, we selected the mealtime situation as the first step of the co-creating process toward a society that coexists with robots.

This research was approved by the Ethics Committee of the University of Tokyo, and all participants signed the informed consent before the experiment.

### 4.3 Experiment 1: Elderly people

#### 4.3.1 Experimental setup and participants

**FIGURE 3:**
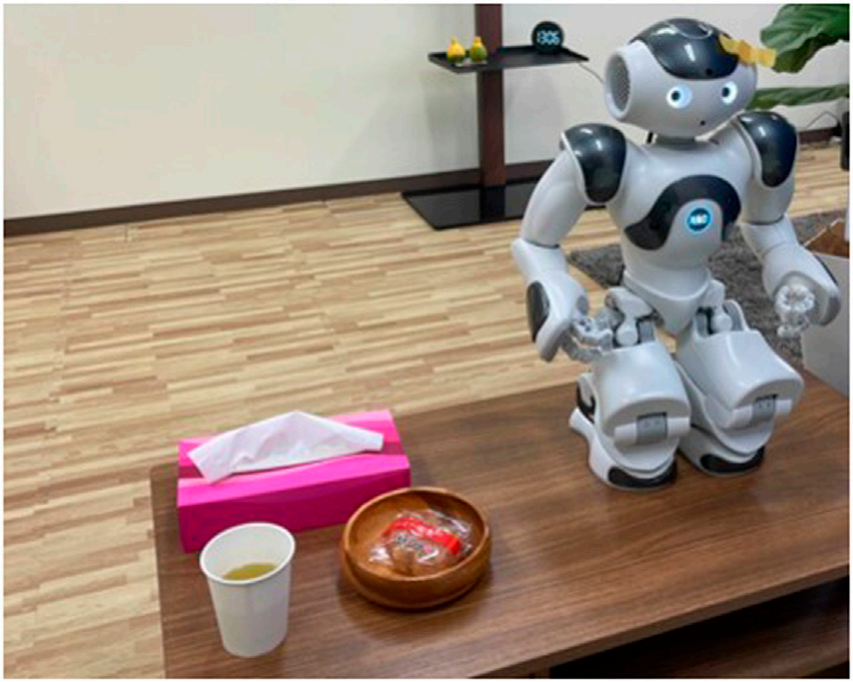
Experimental settings in Living Lab.

As the first step of the co-creation method for the developed robot dialogue interaction system, we conducted a small experiment with elderly people. A total of 12 participants (2 male and 10 female) interacted with NAO while having a snack and drinking tea in a Living-Lab environment (Figure 3). The dialogue situation of the experiment was to talk about the menu for the next meal while checking health status during snack time. The experiment was conducted in a one-on-one setting, and the conversations lasted approximately 5 minutes.

#### 4.3.2 Opinions from participants and discussion

Many of the participants appeared to enjoy interacting with the robot, and some stated that the conversation felt more natural than they had anticipated. Through the experiment, we were able to obtain a variety of useful insights including suggestions for improvement to the robot system from points of view that developers alone could not be aware of. For example, the speed at which the robot speaks was a little fast for some elderly people. It is thought that the realization of a robot system that responds to individual preferences and cognitive abilities is required. In addition, some participants became fed up with being asked the same questions repeatedly due to the speech recognition failure, and some felt as if they were being led through by too many questions from a robot since the conversation in this experiment included many questions to check health status. In human conversation, it is known that self-disclosure, telling other people about themselves, contributes to building relationships (Sprecher and Hendrick, 2004). In human–robot interaction, it is also suggested that self-disclosure of positive content from the robot has good effects on not increasing anxiety about the robot (Nomura and Kawakami, 2011). From these findings, it is inferred that the robot’s self-disclosure about matters related to the content of the questions for knowing about the user is an important element in the dialogue interaction with robots.

### 4.4 Experiment 2: Working-age people and children

#### 4.4.1 Experimental setup and participants

**FIGURE 4:**
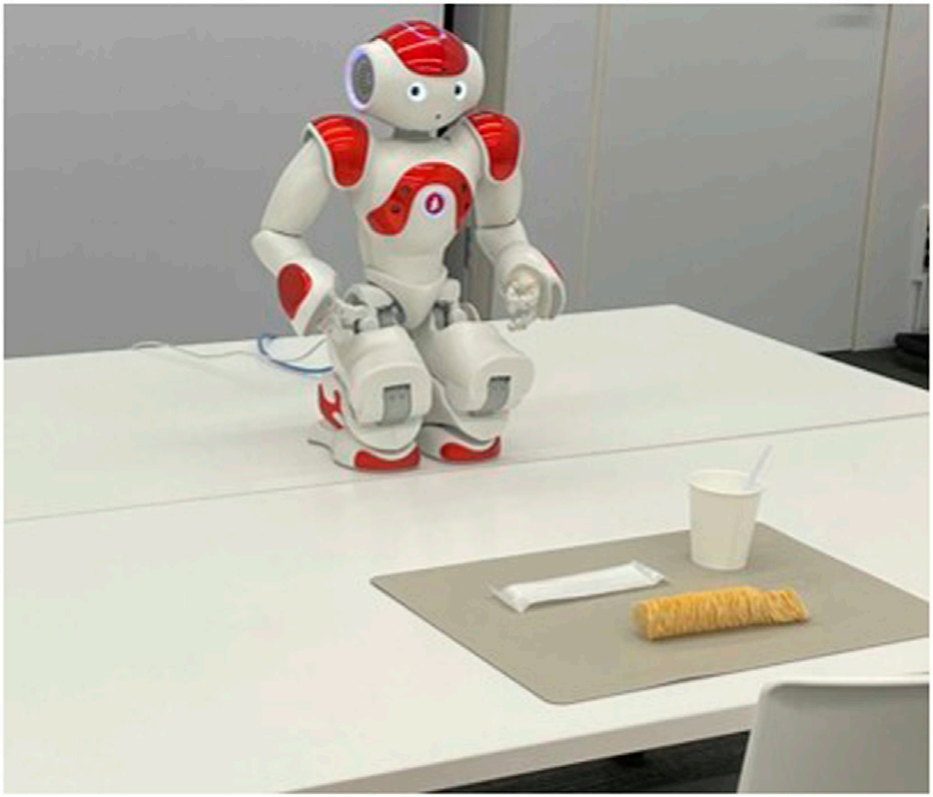
Experimental settings in a science museum.

Next, we conducted an experiment with working-age people and children. The participants were recruited at a science museum. A total of 37 participants (21 male and 16 female, between the age of 8–60 years) were included in the analysis. All participants were served potato chips and water (Figure 4). The experiment was conducted in a situation where the participants chatted over a snack, and the chatting contents included the food topics such as the taste of potato chips, and idle talk such as favorite sports. The experiment was conducted in a one-on-one setting and lasted for approximately 10 min. After the experiment, the participants answered the questionnaire that contained the following Q1–15. Each question was answered on a five-point scale: from 1 (“I do not think so at all.“) to 5 (“I think so very much.“).

Q1 Did you enjoy eating more than when you eat alone?
Q2 Did you enjoy eating more than when you eat with a person who meets for the first time?
Q3 Did you enjoy eating more than when you eat with your family or a friend?
Q4 Did you think the food was more delicious than when you eat alone?
Q5 Did you get along with the robot?
Q6 Did you feel nervous when eating with the robot?
Q7 Did you get tired when eating with the robot?
Q8 Did you feel bored when eating with the robot?
Q9 Did you feel embarrassed when talking to the robot?
Q10 Did you feel the robot to be a disturber during eating?
Q11 Did you think the speaking of the robot was natural?
Q12 Did you think the robot moved smoothly?
Q13 Did you think the robot’s speaking and gesturing are consistent?
Q14 Did you understand what the robot was saying?
Q15 Did you feel that the robot could understand what you said?

#### 4.4.2 Questionnaire results

**FIGURE 5:**
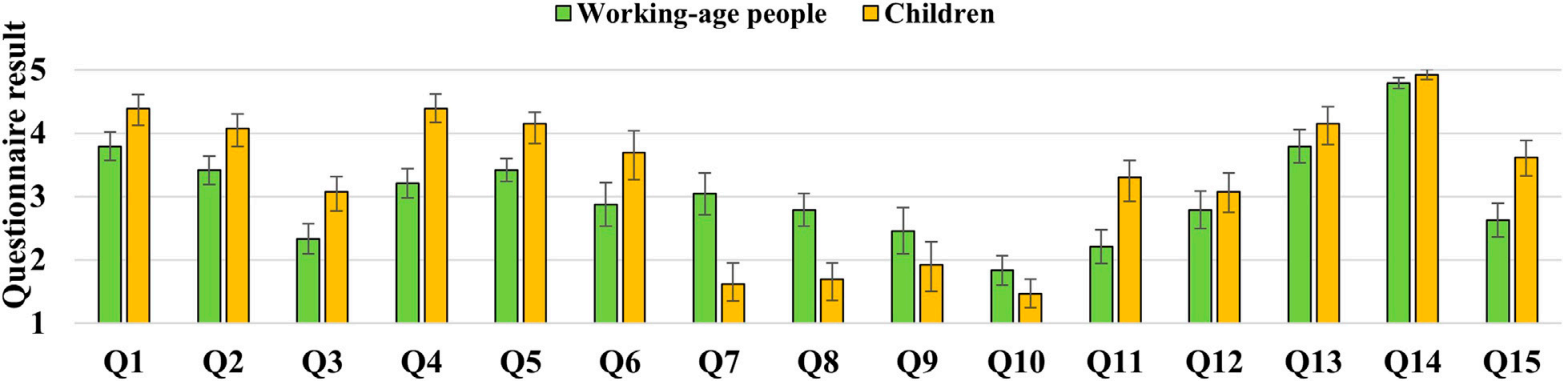
Results of the questionnaires in the experiment with working-age people and children.

Figure 5 shows the mean and standard error of the questionnaire results in each group of working-age people and children. We defined working-age people as those aged 15 and older, and children as those under 15. There were 24 working-age participants (11 male and 13 female) and 13 children (10 male and 3 female).

Tables 1, 2 show Pearson’s correlation coefficients for the relationship between the items regarding the robot’s speaking and gestures (Q11, 12, and 13) and the other items in the questionnaire within the working-age people and children, respectively. We excluded Q14 from the correlation evaluation since most participants answered 5 or 4.

**TABLE 1:** Pearson’s correlation coefficients of questionnaire answers among working-age people.

	Q11	Q12	Q13
Q1	0.001	−0.056	−0.032
Q2	0.244	0.249	0.095
Q3	0.097	0.120	−0.126
Q4	−0.001	−0.104	−0.147
Q5	0.189	0.071	0.198
Q6	0.450	0.590	0.365
Q7	−0.108	−0.107	0.316
Q8	−0.405	−0.242	0.080
Q9	0.353	0.492	0.402
Q10	0.055	0.138	0.155
Q15	0.538	0.416	0.132

**TABLE 2:** Pearson’s correlation coefficients of questionnaire answers among the children.

	Q11	Q12	Q13
Q1	0.407	0.483	0.017
Q2	0.215	0.198	−0.010
Q3	0.092	0.310	−0.009
Q4	0.509	0.513	0.199
Q5	0.549	0.604	0.042
Q6	0.009	−0.258	−0.283
Q7	−0.344	−0.483	−0.302
Q8	−0.653	−0.516	−0.313
Q9	−0.472	0.109	0.169
Q10	−0.534	−0.403	−0.170
Q15	0.727	0.698	−0.0809

#### 4.4.3 Opinions from participants and discussion

In this experiment, based on the findings from Experiment 1, when the robot asked a question and the participant responded, the robot tried to express the impressions and opinions of the response from the participant and disclose its own preferences. An example of the dialogue between the robot and the participant in the experiment is shown in Table 3. Some participants commented positively that it was fun to talk with the robot and the robots seemed to be affable. However, others said that they did not feel like they were having a conversation because they were only answering the questions from the robot, or that there was a lot of prepared talks or parroting back. It was considered necessary for the robot not only to speak its predetermined preferences and ideas but also to expand topics according to the acquired knowledge through communication with humans and to change its own opinions.

**TABLE 3:** Example of the conversation during experiment 2.

Speaker	Conversation
Robot	Do you think the saltiness of the potato chips is good?
	Is it enough?
Human	Yes, I think it is good
Robot	That is good
	I think it is delicious with just the right amount of salt
	The salt flavor is delicious
	I want to know what other flavors of potato chips you like
Human	Um, consomme
Robot	Sorry, I did not hear you well
	May I ask you to speak out loud again?
Human	Consomme
Robot	I see. I think the consomme flavor is also delicious
	But I like a little spicy flavor the best

In addition, in this experiment, even if the robot failed to understand human speech due to speech recognition or dialogue processing failures, it was limited to listening back only once, and if it continued to fail to understand more than once, it responded in a way that seemed roughly consistent with whatever the human had said, and presented the next question or another topic. As a result, there were fewer occasions when participants became fed up with hearing back from the robot. Some participants also commented that even among humans, if they feel they are not communicating very well with the other person, they sometimes ignore the misunderstandings and continue chatting, especially in the case of idle talk. Realistically, it is difficult to achieve perfect speech recognition accuracy for robots, so it is necessary to strike a reasonable balance between deepening the robot’s understanding through accurate comprehension of speech content and allowing the dialogue to continue somehow. For example, when the robot tries to know about the user through dialogue interaction but it is not going well, if the information is not immediately necessary, the technique of reconfirming at a later date is also considered useful (e.g., I am sorry to forget, but what is your favorite food you told me yesterday?)

Some people said that they would be happy enough to just have a robot listen to them and that they would rather share their talk and get empathy from the robot than talk through the questions from the robot. Another participant also suggested that it would be good for robots to make humans feel the value of their existence, based on the idea that in human relationships, it is important to be needed by the other person. From these considerations, it is suggested that robot behavior that satisfies the human need for approval is also important in building a good relationship between humans and robots.

The correlations of questionnaire results among children showed a positive correlation between the smoothness of gestures and familiarity with the robot. Although not significantly different, there was a positive correlation of more than 0.4 between familiarity with the robot and the naturalness of speaking, and a negative correlation of less than −0.4 between the negative feelings that the robot was a disturber and the smoothness of conversation and gestures. In addition, in Q1 and Q4 which related to the eating experience, such as the enjoyment and taste of the meal, there was a positive correlation of 0.4 or higher for both conversation and gestures, though there were no significant differences. In contrast, however, no high absolute correlations of 0.25 or higher were found for these items in adults. Furthermore, among adults, ratings of whether the robot’s speaking and movements were natural and smooth were positively correlated with nervousness, and the smoothness of the robot’s movements was positively correlated with embarrassment. An adult participant commented that he became emotionally involved with the robot, suggesting that different age groups may have different perceptions of the robot. From these factors, it is suggested necessary to consider strategies for robot speaking and gestures for different age groups, such as creating an affable atmosphere for adults to reduce their nervousness and shyness about interacting with the robot at first.

As for the technical issues, there were opinions that, in the case of speech recognition in Japanese, the accuracy of picking up sounds is required because “un” means “Yes” and “uun” means “No.” A participant said that it may be necessary to judge whether the utterance is positive or negative from the tone of voice and accent as well as words and to read facial expressions. It is thought that a multimodal understanding of intentions using not only voice but also the camera and sensors of the robot is required.

## 5 Conclusion

In this article, we explained a ROS-based dialogue interaction system at first. The system integrates automatic speech recognition, natural language processing, and dialogue management which are the major technologies for voice interaction, and we incorporate the gesturing of the robot in the system. Using the interaction system, we conducted two experiments with elderly people, working-age people, and children as the initial phase of the co-creation process. Through the experiment, we could get many useful insights from the participants’ reactions, questionnaires, and opinions that the robot is required to respond in a way that is not one-sided, that the robot is required to not only accurately grasp the content of the conversation but also to have the skills to successfully continue the conversation, and that the perceived smoothness of the robot’s speaking and gesturing affects the increase of nervousness and embarrassment among the working-age population.

In future work, based on these findings from the experiments, we would like to conduct specific studies on issues such as how to solve the problem of speech recognition accuracy with interaction methods, how to help the robot understand human speech more deeply, and to what extent the robot is required to express its own opinions. It is also necessary to investigate the behavior of robots so that humans feel comfortable living together according to their age. In the technological aspect, we plan to connect the interaction system with various databases and ontologies to enhance its dialogue capabilities and make it usable for various situations. We also want to combine the system with additional recognition modules, such as image recognition and emotion recognition, since we believe that multimodal information creates more natural interaction between robots and humans.

The final goal of this research is to make robots become good partners for humans and more socially acceptable. In order to achieve this goal, we would like to continue the cycle of improving the robot system by repeating the co-creation process. Despite the several limitations such as the type of the robot and the country in which the experiments were conducted, we believe that this research provides useful initial findings and insights into the robots as great companions.

## Data Availability

The original contributions presented in the study are included in the article; further inquiries can be directed to the corresponding author.
